# Optimized recycling of e-waste and tire rubber into sustainable polyurethane composites with enhanced compressive performance

**DOI:** 10.1038/s41598-026-50153-6

**Published:** 2026-05-28

**Authors:** Vinoth Kumar Selvaraj, Jeyanthi Subramanian, Aadithya Narayanan S, Nitesh Verma Kannammaraju

**Affiliations:** 1 Interdisciplinary Research Center for Construction and Building Materials, King Fahd University of Petroleum & Minerals (KFUPM), Dhahran, 31261 Saudi Arabia; 2 School of Mechanical Engineering, Vellore Institute of Technology, Chennai, 600127 Tamil Nadu India; 3 School of Mechanical Engineering, Vellore Institute of Technology, Amaravati, 522241 Andhra Pradesh India; 4 Department of Mechanical Engineering, University of Michigan, MI 48109 Ann Arbor, USA

**Keywords:** Sustainable composites, E-waste recycling, Rubber tire waste, Polyurethane foam waste, Compressive strength, Circular economy, Energy science and technology, Engineering, Materials science

## Abstract

Research on reusing industrial and electronic waste in functional composite systems has been spurred by the growing need for sustainable engineering materials. This study used methylene diphenyl diisocyanate (MDI) as a binder to reinforce three recycled fillers: waste rigid polyurethane foam (WRPU), rubber tire waste (RTW), and waste printed circuit boards (WPCB). Response Surface Methodology (RSM) optimization revealed that the optimal composition was 6 wt% WRPU, 3 wt% RTW, and 7.58 wt% WPCB. With a maximum compressive strength of 38.987 MPa, this optimized formulation showed significant improvement over the unreinforced matrix (14.128 MPa). The mechanical performance was highly precisely validated by numerical simulation using ANSYS Workbench, with a deviation from the experimental results of only 0.75%. FTIR and thermogravimetric analysis (TGA) indicated enhanced interfacial interactions, improved compatibility and thermal stability up to 550 °C, while high-resolution scanning electron microscopy (HR-SEM) verified uniform filler distribution. By enhancing interfacial bonding and stress transfer efficiency, the synergistic interaction of WRPU, RTW, and WPCB clarified the structure–property relationship controlling composite performance. These results support a sustainable circular economy by showcasing the potential of recycled polyurethane composites for lightweight, compressive load-bearing applications in automotive non-structural components.

## Introduction

In the last few years, materials testing has emerged as an indispensable and recognized discipline for assessing the physical and mechanical characteristics of materials, from powders and raw materials to parts and end-use composite products. Compression testing is a frequently performed mechanical testing procedure that provides important information about the response of materials under a compressive load. Compression testing is a large component in material selection in many industries, including aerospace and automotive industries, as well as construction^1^. In the actual test, a specimen is placed between two plates or grips of a testing machine, and a compressive force is applied until the material deforms to a predefined amount or fails. The specimen is interpreted using a deflectometer and/or an extensometer to measure the strain, typically strain. The main purpose of compression testing is to assess the response of materials subjected to compressive loading, or what parameters like stress, strain, and deformation it creates^2–5^. Throughout the past few decades, several researchers have investigated the compressive behavior of composite materials due to its significant role in affecting the structural integrity of a lightweight structural design. Many of these studies include internal failure modes such as fiber kinking, matrix cracking, delamination, and micro-buckling, which occur under compressive loading^6–12^.

In the last couple of years, numerous studies have focused on gaining an understanding of the compressed behavior of all materials under static loading. These studies have been showing that composites may develop internal failure mechanisms (e.g., matrix shear, fiber kinking, micro-buckling, delamination) where composites can appear undamaged but can lose significant load-carrying capability. Understanding which damage mechanisms can evolve is important to make accurate predictions for composite failure modes and also to minimize materials^13–20^.

In this research, the production of a sustainable composite with three forms of recycled fillers including rigid polyurethane foam waste (WRPU), ground tire rubber (RTW), and discarded printed circuit boards (PCBs) is studied. Each of these fillers has specific advantages, contributing in separate ways to the mechanical system performance of the final product. Polyurethanes (PU) are produced by polyaddition reaction of diisocyanates and diols^21^. Thus, the production of PU is dependent on petrochemical feedstocks. PUs is well known for being energy efficient, durable, cost-effective, and flexible to use, making them some of the most commonly used materials in all industrial sectors. PU foams are categorized by density as semiflexible, flexible, semirigid, and rigid. Soft PU foam has superior specific elasticity, elongation, along stability to solvents and chemicals. These characteristics allow polyurethane foams to fulfill a wide range of applications involving cushioning and insulation. Flexible PU foams are used for reclining seats, headrests, and soundproofing in automobiles^22,23^. Rigid polyurethane (PU) foam is well noted for its excellent mechanical strength, thermal insulation, and acoustic insulation^24^. It is a primary role in thermal insulation in construction as well, for thermal insulation of wall insulation, roofs, and floors, often as part of Structural Insulated Panels (SIPs). Because of its lightweight construction and ability to support and insulate, rigid PU foam is also used in automotive and aerospace applications^23^. Polyurethanes (PUs) can be formulated with very flexible networks that can be suited to plastic substrates, in contrast to automotive coatings which are formulated to be tough, strong, and resistant to impacts from road debris and a range of weather^25,26^.

The swift growth of the automotive industry has caused an incredible increase in the number of vehicles in the world, and has also led to severe environmental impacts. Recent studies have further highlighted the adaptability of PU foam systems for advanced composite applications and sustainable material development^27–29^. Besides CO₂ emissions, which are well known, a key issue related to the end-of-life for vehicles refers to the substantial buildup of enormous quantities of end-of-life tires^30^. A tire is an intricate, engineered product that has one basic function: to turn the energy supplied by the engine into usable power at the road surface. Rubber constitutes approximately 45% of the weight of the tire; other materials include steel belts, fabric plies, performance-focused fillers, and various chemicals. The rubber for tires comes in both natural and synthetic forms. Natural rubber (NR) is valued for a combination of fatigue resistance and tear strength, and natural rubber materials are enhanced with synthetic rubbers – in particular, butadiene rubber (BR) and styrene-butadiene rubber (SBR) exhibit appropriate performance characteristics such as rolling resistance, load bearing, traction, fatigue and durability. Truck tires are made with significantly more natural rubber than a passenger car tire because trucks represent a significantly larger load and more time in operational service, hence the need for the durability and fatigue resistance that natural rubber provides^31^. Since tires are manufactured in bulk and cannot decompose, it creates a noteworthy risk to our environment. Governments and environmental organizations have made efforts to develop regulations to promote environmentally safe recovery and recycling of tire waste^32^. The most effective method includes environmentally friendly practices to minimize the consumption of virgin raw materials and help transform waste tires into higher-value industrial output^33^.

Printed Circuit Boards (PCBs) are the structural base and functional core of the majority of electronic devices^34^. Printed circuits boards (PCBs) are ubiquitous in many electronic devices ranging from ordinary 3 C categories, e.g. computers, communication devices, consumer electronics, etc., to home appliances and automotive electronic systems^35^. They are typically constructed of epoxy laminates and fiber-reinforced composites. It is common for even small manufacturing defects to create catastrophic failures in the end products. Therefore, it is also important to produce PCBs precisely and to high-quality standards; reliable electronic assemblies can only be developed in high-quality basic materials^36^. The growing global need for electronics has made the development of reliable and efficient methods for the detection of PCB defects during manufacturing an even more important requirement^37^.

WRPU, RTW, and WPCB are three recyclables that bring their own strengths, including low weight, energy dissipation, and structural stability^38^. Because these materials have different compositions and structures, a prototyped processing technique is crucial for ensuring these composite waste materials are compatible and processed uniformly. In this work, the goal was to use mechanical recycling methods to allow for these materials to be incorporated more readily. The significant increase in PU waste has raised concerns regarding the disposal of polyurethane and the environmental consequences^39^. Specifically, in the Asia-Pacific region, production of PU material was estimated at 11.5 million tons per year, and forecasts to exceed 15.5 million tons by 2019 ^40^. PU foam waste can typically be recycled in two ways: through physical recycling and through chemical recycling^41,42^. Physical recycling involves reusing polyurethane (PU) foam in its original form while remaining intact chemically, which represents a practical and economical option when dealing with PU waste management. This process is often thought to be quasi-resourceful. One example of physical recycling is mechanical grinding, or shredding, where PU foam is processed into small lumps or pieces. These lumps can be used as fillers or further processed into rebound foam for uses such as carpet underlays or insulation. Regrinding can be accomplished by pulverizing the foam into powder and then blending it with virgin PU to form new layers of foam—primarily for low-density foam applications such as seat cushions and padding. In comparison, chemical recycling uses more sophisticated techniques of glycolysis, hydrolysis, aminolysis, and thermochemical processing, as well as biodegradation. These processes can digest PU into its fundamental unit value to recover some important raw materials such as polyols, which can be reused to manufacture new polyurethane products^22,43^. With mechanical recycling of PU waste, the discarded PU is transformed into small particles and finer powder sizes and ranges, which can be microns in size and a few millimeters in size^44^. The recycled PU particles can be used as inert fillers, or sometimes reactive diluents, in polyol formulations to create new PU systems^40^. As stated by the European Tyre and Rubber Manufacturers Association, mechanical treatments—such as shredding and grinding—account for about 87.5% of the methods to recycle rubber waste or products from tires, including PU foams. In order for re-use and to be both efficient and reliable recycling process, it is important that we classify the rubber waste depending on the source before processing. The classification allows us to make process changes to improve the quality of the final recycled product. The qualities of ground rubber such as particle size and surface texture can vary with respect to the method the rubber is ground and the conditions of processing. Waste tires are generally converted into three main output materials: steel fibers, textile reinforcements, and ground tire rubber (RTW)^45,46^. Ground tire rubber (RTW) is then reused as a filler in many composite applications, like those studied in the current study^47^. Electronic waste (e-waste) is comprised of broken, outdated, and/or non-working electrical and electronic devices that are no longer in use and are to be recovered, recycled or disposed of. This covers a wide range of devices, for example, components of computers and other peripheral equipment^48^. From all recycling methods, mechanical recycling is a prevailing one for the processing of printed circuit boards (PCBs) due to its low-cost, reliable operation and thermal efficiency^49^. Additionally, recent studies have concentrated on altering natural rubber composites to enhance their qualities for environmentally friendly uses, offering insightful information about rubber recycling^50–52^. The normal recycling method starts by separating printed circuit boards (PCBs) before moving them to smelting plants which extract valuable metals including gold and silver and copper^53^.

The recovery process of PCBs regularly encounters multiple obstacles for efficient material retrieval^54^. The main issue with achieving proper delamination for component separation is that it requires ultra-fine fragmentation which proves both expensive and consumes excessive energy^55^. Despite these challenges, the current standard practice involves disassembling and sorting PCBs before sending them for metallurgical processing, where partial material recovery is carried out^56^. The mechanical recycling process of printed circuit boards (PCBs) begins with shredding to create easier handling conditions and reduced volume and better surface contact for improved material extraction. After component shredding ends a critical factor emerges about polyurethane adhesives which hold PCB layers together as a single composite. The adhesives demonstrate essential value in industrial and domestic environments since they produce enduring bonds and outperform numerous other adhesive types in versatility. The thermosetting properties of polyurethane adhesives lead to the creation of bonds which remain in place indefinitely. The reaction between isocyanates and hydroxyl groups in these adhesives produces urethane linkages as their typical component. Methylene diphenyl diisocyanate (MDI) stands as a widely used isocyanate in composite bonding because it exhibits high reactivity together with hygroscopic properties to draw moisture which leads to strong urethane bond formation^57^. The manufacturing process of particleboard (PB) and high-density fiberboard (HDF), and medium-density fiberboard (MDF) extensively employs polyurethane adhesive since it improves both physical and mechanical attributes of finished panels^58^. MDI adhesive functions as the key bonding agent between recycled PU foam and ground tire rubber and shredded PCBs which requires determining proper filler ratios to achieve optimal composite performance. Optimization techniques are utilized to discover optimal filler ratios that lead to superior composite performance. The Response Surface Methodology (RSM) serves as a widely adopted and powerful experimental optimization technique. The Response Surface Methodology (RSM) provides statistical and analytical tools for evaluating problems with responses that depend on multiple variables. Researchers use RSM to generate empirical models through planned experimental sequences that discover exact input parameter conditions which optimize desired outcomes. The research employed RSM to find the best filler mix which maximizes compressive strength before creating a composite with improved characteristics^59^. Therefore, the primary objective of this study is to present the initial examination of compressive properties in composites made from WRPU and RTW combined with PCB waste using MDI adhesives. The study achieves additional novelty through its use of Response Surface Methodology (RSM) to optimize compressive strength in recycled composite materials.

## Experimental procedure

### Materials

Rigid Polyurethane Foam Waste (WRPU) was collected from ASL and DRDO Hyderabad in Telangana and then physically reprocessed by grinding into a fine powder with an average particle size of 200–300 μm. The Waste Printed Circuit Boards (WPCBs) were gathered at VIT Chennai Electronics Laboratory and then mechanically processed into particles measuring 30–40 μm. Ground Tire Rubber (RTW) waste was acquired from VIT Chennai Center for Advanced Material, and its particles were pre-shredded to sizes between 100 and 150 μm. The adhesive Flexibond based on MDI, was bought from Manali Petrochemicals Limited, located in Chennai, India. The study used distilled water, which was supplied from the Chemistry Laboratory at VIT Chennai.

### Preparation of the composite

**Fig. 1:**
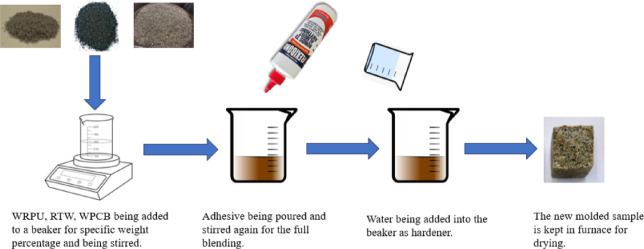
Schematic diagram of the manufacture of the composite.

Rigid Polyurethane Foam Waste (WRPU) and Waste Printed Circuit Boards (WPCBs), and Ground Tire Rubber (RTW) were mixed in predetermined proportions to create a sustainable recycled composite according to the table. The compressive properties of the recycled polyurethane composites were assessed in accordance with ASTM D695 - Standard Test Method for Compressive Properties of Rigid Cellular Plastics. A beaker received the measured fillers, which were then stirred for 10 min at 150 rpm to obtain an even distribution. The adhesive used for this project was chosen from bio-based MDI to enhance environmental sustainability. The filler mixture received 30 g of adhesive, which was then stirred for 15 min until every surface received complete coverage. A hardener solution consisting of 15 g of distilled water, which matched 50% of the adhesive weight, was added for 5 min of blending. The liquid mixture entered a silicone mold, which contained dimensions of 30 mm by 30 mm with a depth of 30 mm. A pressing tool designed to match mold dimensions was used to compact the blend with a pressure of 4 MPa before leaving it to cure at room temperature throughout the night. After 24 h, the cured sample was extracted for hot air oven treatment at 100 °C, which lasted for 6 h to remove remaining moisture. The individual fillers’ characteristic properties appear in Table 1, while Table 2 lists the exact filler percentages used in the MDI binder. Figure 1 shows the schematic diagram of the manufacture of the composite.

**Table 1:** Details of Reinforcement.

Particle	Size (µm)	Density (g/cm^3^)	Melting Point (℃)
Rigid PU foam	200–300	0.03–0.06	120
PCB waste	30–40	1.8–2.2	180
Tire rubber scrap	100–150	1.1–2.2	270

**Table 2:** Different levels of fillers mixed with Flexibond.

Code	Parameters	Level -2	Level -1	Level 0	Level 1	Level 2
A	WRPU (wt%)	6	12	18	24	30
B	WPCB (wt%)	2	4	6	8	10
C	RTW (wt%)	3	6	9	12	15

Note: Based on preliminary manufacturing trials and literature values, specific ranges of WRPU (6–30 wt%), WPCB (2–10 wt%), and RTW (3–15 wt%) were chosen. Below these concentrations, the WRPU, WPCB, and RTW reinforcement materials were not sufficiently bonded together, and above these concentrations the samples compromised processability and integrity. The coding system has five levels from − 2 to + 2 as noted in Table 2, which indicates the minimum to maximum workable values for these range values.

### Compression test setup

The research evaluates how foam specimens react to compression through a Universal Testing Machine (UTM). The Shimadzu AG-Xplus 50kN UTM compression platens receive square foam specimens with specific dimensions and density measurements. The compressive properties of the composite specimens were evaluated by means of ASTM D695 - Standard Test Method for Compressive Properties of Rigid Plastics. The samples receive proper alignment for uniform load application and stability when undergoing testing. The machine undergoes calibration before testing, while the load cell and displacement sensors receive accuracy verification. The testing protocol defines specific crosshead speed parameters for performing compression tests. The testing process records important parameters, including compressive force alongside deformation and stress–strain response. The research evaluates composite foam material effectiveness through analysis of collected data, which produces maximum compressive strength and deformation behaviour. Stiffness-related parameters obtained from the stress-strain response are reported as apparent values due to the nonlinear deformation behaviour of the composites.

### Response surface methodology (RSM)

RSM represents a statistical technique which evaluates how independent variables affect dependent responses through multiple steps of analysis^60^. The main concept behind RSM consists of designing specific experimental procedures to find optimal operational parameters. RSM enables the measurement of variable interactions which improves the precision of process optimization when compared to traditional trial-and-error approaches. Researchers can apply Central Composite Design (CCD) to create second-order polynomial models that serve as approximations when dealing with multiple anticipated factors. The second-degree model enables target response optimization through methods that seek maximum, minimum or specific value attainment.

### Computational simulation

The finite element analysis was carried out with ANSYS Mechanical (a commonly utilised tool for structural simulation), using automation to support the modelling workflow of simulation through the use of user-defined scripts^61^. ANSYS created a simplified homogeneous cube model of the composite material for the efforts. The incorporation of fillers was accomplished using an effective method of homogenisation, but individual variation in particle size, multiple phases and dispersion and interfacial effects were not modelled directly. This simplified modelling approach was utilised to reduce the complexity of calculations for qualitative comparison of trends from experimentation without replicating the composite microstructure in detail.

### Characterization technique

Using a high-resolution scanning electron microscope (HR-SEM) (Zeiss EVO10), the morphology of the recycled fillers and the fracture surface of the composites were analyzed. Imaging was performed by a LaB₆ electron gun operated in secondary electron (SE) mode, using the accelerating voltage of 30 kV. Reference samples were coated with a 0.5 μm gold layer to eliminate charging prior to the analysis. A spectrometer manufactured by Agilent called the Cary630 FTIR was utilized to identify the functional chemical groups in the sample. Data were collected over the range of 4000 –400 cm^− 1^ at a resolution of 4 cm^− 1^. This was performed to determine if there was evidence for the formation of a urethane bond. The thermal stability of the sample was examined by TGA (thermal gravimetric analysis) of the composite materials using a TA Instruments SDT Q600. For this test, the composites were warmed from 30℃ to 900℃ at a relative rate of 10℃/min in a nitrogen gas chamber (50 mL/min) using a 10 mg sample in an alumina crucible.

## Results and discussion

### High-resolution scanning electron microscope images

**Fig. 2:**
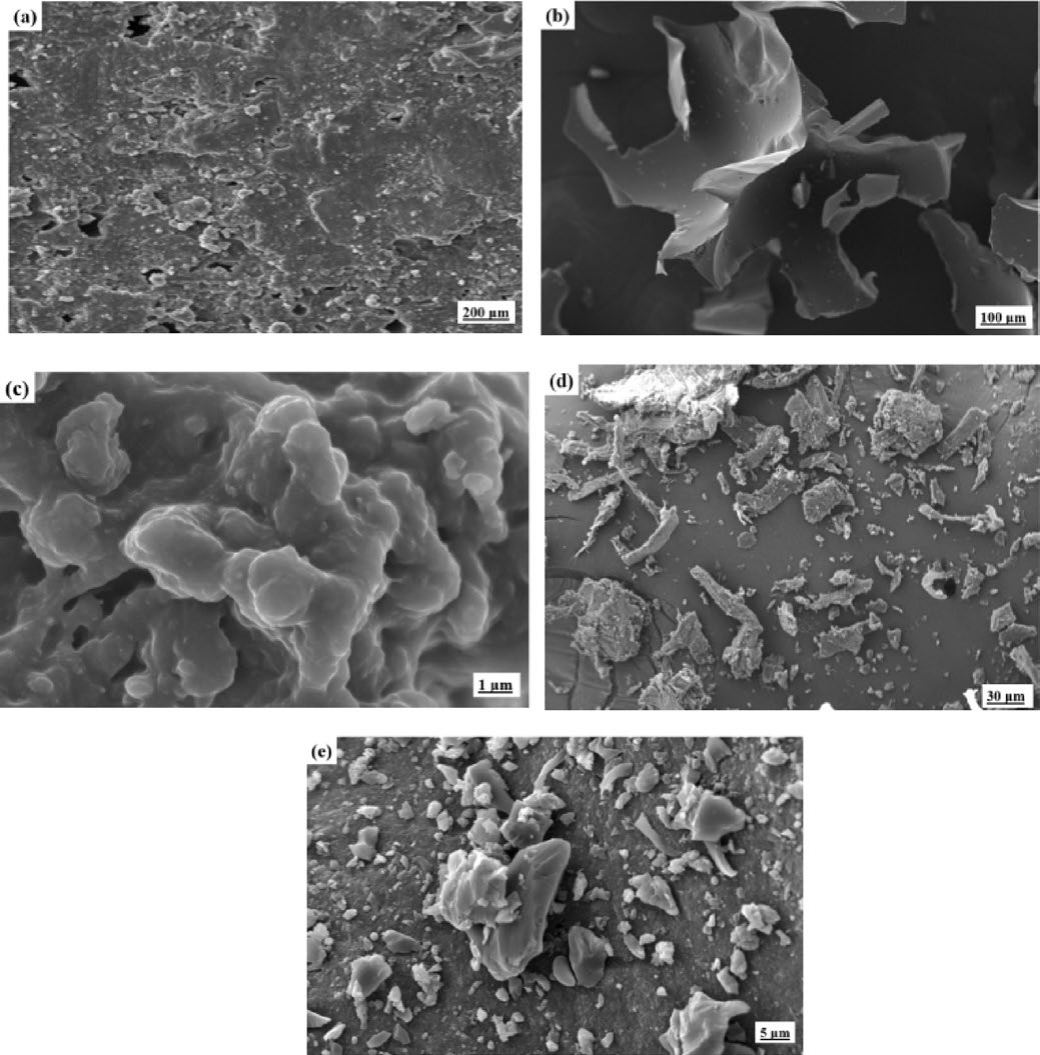
HR-SEM characterization of (**a**) MDI based sample without filler, (**b**)WRPU, (**c**) RTW, (d)WPCB, and (**e**) MDI based sample with filler.

High-Resolution Scanning Electron Microscopy (HR-SEM) is an advanced imaging method used to investigate the surface topography and microstructure of materials at a very high resolution, sometimes at less than 1 nm ^62–65^. HR-SEM utilizes electron beam technology, in which a source of electrons, typically generated by an electron gun, generates a focused and narrow beam of electrons, which is directed, and focused onto a defined section of the sample using electromagnets. When the electron beam interacts with a material’s surface, it creates nanoscale images at high resolution. One of the clear advantages of HR-SEM is the excellent view of the surface structure. HR-SEM is an increasingly important characterization tool in material science for production-quality control, design of new electronic materials, and failure analysis. In this study, the surface features of each constituent material noted by the SEM observations exhibit different characteristics. When viewed at lower power, the unmodified MDI structure shown in Fig. 2(a) shows its visible cellular architecture and exhibits voids and gas bubbles due to no reinforcement. Gas evolving reactions, as in this case, typically (not always! ) result in the formation of micro-pores during the formation of polyurethane. Figure 2(b), shows the surface of a rigid polyurethane foam material the authors would use as a common waste material. The rigid foam material demonstrated a brittle fracture with sharp edges and flakes, indicating the structural brittleness nature of this waste foam. Although the material appears dense, the surface morphology indicates an uneven surface texture that was due to the non-uniform cellular structure of the rigid foam, which resulted into surface irregularity. It was indicated from previous work^66,67^ that the rigid PU foams, had, closed-cell morphologies. In Fig. 2(c), the surface of the RTW shows a highly irregular and wrinkled texture which would be typical of vulcanized rubber surfaces filled with carbon black, a common filler for the tire industry. Figure 2(d) is the SEM image of the shredded waste PCBs which showed rough and fibrous, with flake-like textures of the glass fiber-reinforced epoxy material after mechanical wear. Lastly, Fig. 2(e) clearly indicates the microstructure of powdered fillers within the WRPU matrix on the SEM, showing that the powdered forms were evenly dispersed throughout the unmixed foams, showing that there was good mixing and proper reinforcement of the composite. The SEM micrographs were created using multiple levels of magnification to see structural features at many different lengths. Lower magnification demonstrated the macroscopic distribution of voids and defects, whereas higher magnifications evaluated the microscopic interfacial adhesion and filler dispersion.

### Fourier transform infrared test

**Fig. 3:**
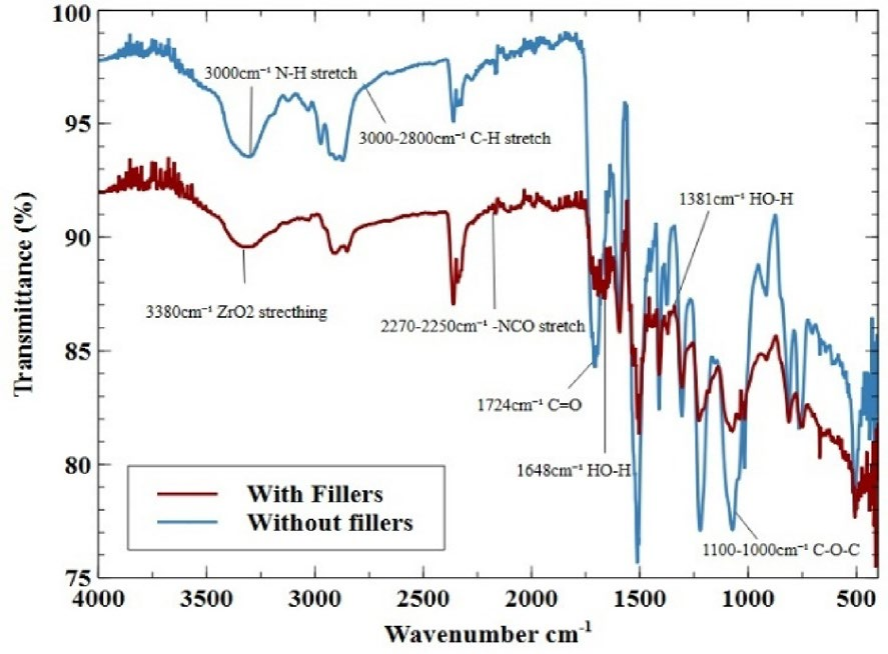
FTIR graph of the sample with and without fillers.

Fourier Transform Infrared Spectroscopy (FTIR) is a typical method to mark organic and inorganic materials based on their absorption of infrared radiation. ^68–70^. When infrared light penetrates some sample, it is absorbed at specific wavelengths and certain molecular bonds generally absorb at specific wavelengths resulting in a distinctive spectral signature. FTIR allows any user to identify functional groups, elucidate molecular structures, and analyze the relative changes between vibrational modes of molecules. In order to minimize fluctuations in baseline and reduce interference from the atmosphere, all FTIR readings were made at a controlled laboratory environment. The spectra obtained were subsequently processed to enhance the signal-to-noise ratio. Figure 3 shows that both samples have FTIR spectra that differ greatly in chemical composition^71,72^. The blue curve represents a sample of rigid PU foam dust with MDI in the sample while the red curve is the sample of rigid PU foam dust with MDI and also major filler materials. In the blue spectrum once again, the results of the reacting mixture of methylene diphenyl diisocyanate (MDI) and the rigid PU foam dust resulted in functional groups that were characteristic of this reaction. The wide absorption band in the range of approximately 3200–3500 cm⁻¹, is predominantly due to the O–H stretching vibrations of residual moisture or hydroxyl groups; however, N–H stretching of urethane groups may have a lesser contribution; another strong peak at 1724 cm⁻¹ indicates the stretching of C = O bonds that we would expect from urethane structures. The absorption band around 2270–2250 cm⁻¹ indicates unreacted or improperly reacted isocyanates (-NCO). The weaker peaks at 1648 and 1381 cm⁻¹ only indicate traces of hydroxyl (-OH) groups produced by any residual moisture. Below 1000 cm⁻¹, sufficient signals are observed for aromatic C–C and C–O–C vibrations associated with the MDI structure. The peak at 1381 cm -1 indicates phenolic OH groups, usually present in rubber materials, which appear to take prominence in this system^73^. FTIR analysis profiles change considerably with the addition of fillers, (shown in the red spectrum). New or sharper or additional peaks, such as 3380 cm⁻¹ and 550 cm⁻¹, have emerged. These peaks can be identified as Zr–O and indicate the presence of zirconium-based portions are likely to have a ceramic component commonly found in waste PCBs^74^. The -NCO peak also could be diminished, as it reacts with excess -OH groups from fillers, while the C–O–C stretching region (1100–1000 cm⁻¹) becomes better defined widely depending upon the filler content. It is important to note that the composites being tested were created primarily from the physical mixing of recycled fillers into MDI-based polymers. While changes in peak intensity and shape may be indicative of interfacial compatibility, the FTIR results provide only indirect qualitative evidence for chemical interactions. Minor baseline fluctuations may be caused by residual effects from the atmosphere. Future work should therefore incorporate more sophisticated techniques for characterising surface and bonding, such as XPS or solid-state NMR, to further elucidate interfacial chemistry.

### Thermogravimetric analysis

**Fig. 4:**
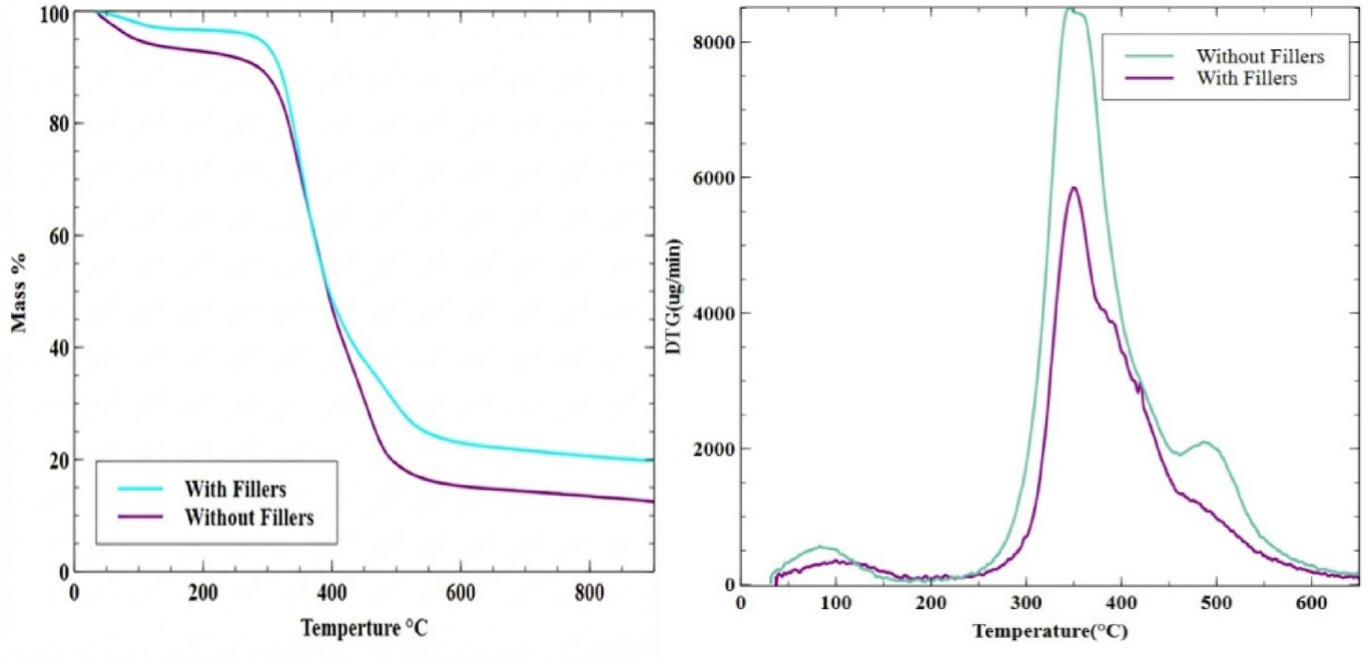
(**a**) TGA curves (Mass%) of the sample with and without fillers. (**b**) DTG curves of the sample with and without fillers.

The sample—established as clean, dry, and compositionally homogeneous—is placed inside a lightweight aluminum crucible, which is attached to the precision balance of the thermogravimetric analyzer. The temperature is then programmed to ramp at a steady rate of 10 °C/min up to a predetermined maximum temperature, in an inert atmosphere maintained with either nitrogen or argon gas. The corresponding mass loss due to thermal degradation process is monitored and reported in Fig. 4a. The unmodified (pure) sample shows an abbreviated and distinct degradation in the temperature range 350–500 °C, which was determined to be indicative of urethane linkages and isocyanate groups breaking down. The composite with fillers started to show degradation more slowly with the thermal degradation temperature range greatly expanded, it suggests that thermal resistance was improved from adding thermally stable materials like silicates, metals and zirconium oxides that corresponded to the PCB fillers, in addition to carbon signatures from tire scraps^75–77^.

The higher residual mass recorded for the filled sample suggests that the added inorganic fillers are heat resistant and contribute to greater char formation, which agrees with the peaks seen on the FTIR spectra at 550 cm⁻¹ and at 3380 cm⁻¹ due to ZrO and other stable oxides found within the fillers. This was further confirmed by the DTG curve (Fig. 4b). The pure PU sample has a strong peak between 370 and 400 °C, indicating that the urethane bonds are rapidly degrading. The filled composite instead showed two smaller peaks suggesting a two-step decomposition where the first peak evidenced where the PU matrix was decomposing, the next peak occurred between 500 and 550 °C suggesting that the organic parts that are found in tire rubber or PCB materials, was breaking down. The thermal decomposition shown by DTG suggests that the fillers present slowed this decomposition process down, as well as led to more char formation, and overall improved thermal stability. Further support of the thermal stability improvements of being filled, is the decreased height of the DTG peak for the filled sample compared to the pure sample, showing further evidence that degradation happens slower^77,78^.

### Experimental analysis

**Fig. 5:**
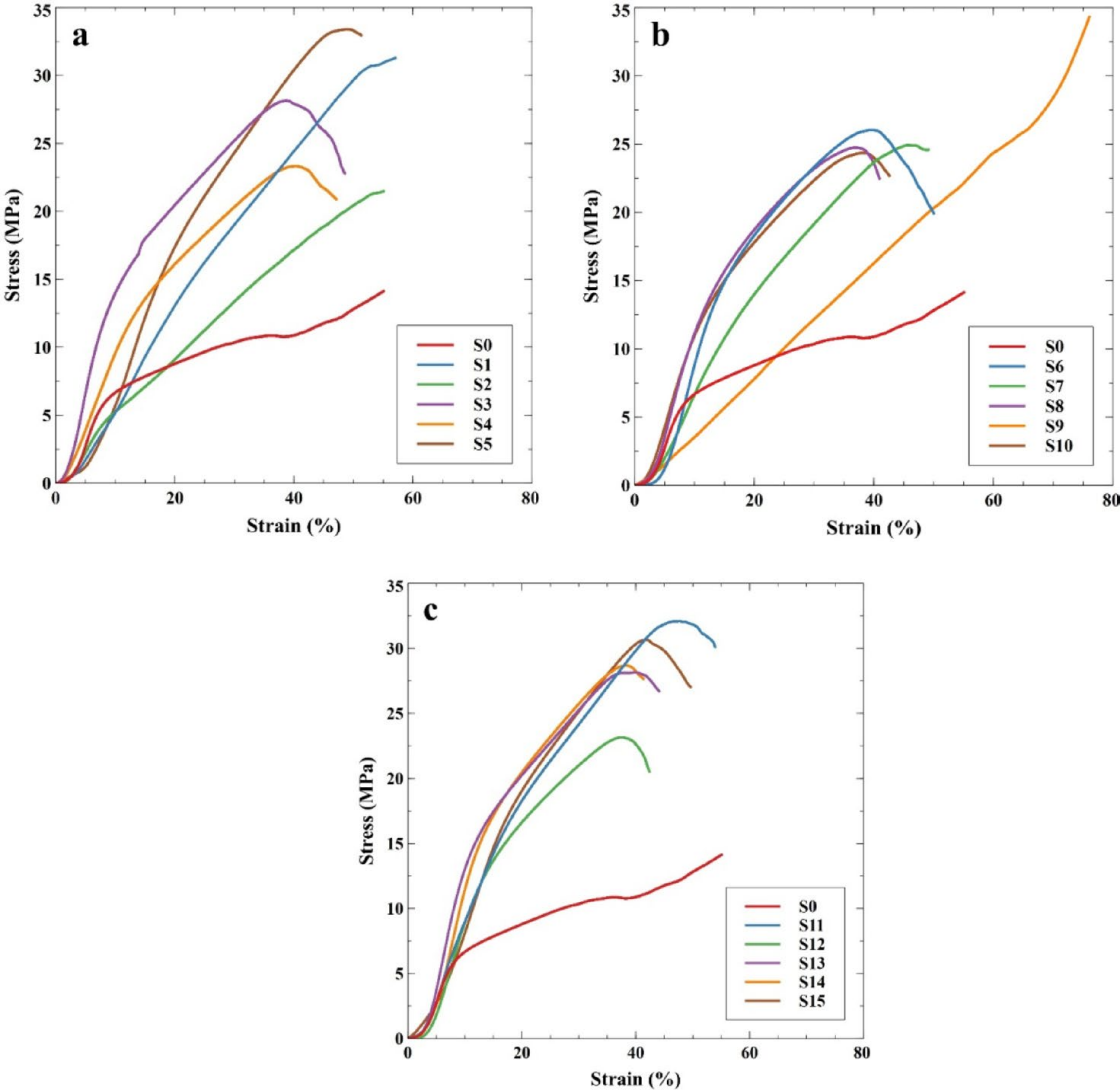
Stress-Strain graph for **a**) Sample S0 - S5 **b**) S0, S6 - S10 **c**) S0, S11–15.

Mechanical properties of WRPU foam composites with RTW and WPCB fillers were evaluated using compressive strength testing. Samples of square disc geometry were subjected to compression tests, and the entire sample group (S0-S20) was tested in order to find an optimized formulation. The mechanical properties of samples S0-S15 were analyzed using stress/strain curves shown in Fig. 5 (a)-(c). S9 has the highest compressive strength (34.310 MPa) at a formulation of 6 wt% WRPU, 9 wt% RTW, and 6 wt% WPCB. The optimized formulation created an ideal balance of stiffness/toughness because of the interaction between the three fillers. WRPU provided a lightweight cellular structure; RTW provided elastomeric properties that helped resist deformation; and WPCB provided a stiff/strong load-bearing structure through glass fiber and metal content. In terms of mechanical performance, the balance described above illustrates the trade-off between increased stiffness and energy dissipative mechanisms. While WPCB is responsible for increasing both stiffness and load-bearing capacity, RTW increases energy absorption when subjected to compressive loading. Therefore, increasing compressive strength does not directly correlate with increasing stiffness, suggesting that optimum formulations achieve improved performance through improved stress transfer rather than solely through elastic reinforcement. The rigid WPCB particle and flexible RTW interaction provides a resistance to deformation while reducing brittle failure, as seen in most rigid foam systems.

From a failure mechanism standpoint, the compressive stress of the returned composite is primarily a function of progressive cell failure, localized filler/matrix debonding and controlled energy dissipation versus catastrophic brittle failure. Excessive rigid filler can cause stress concentrations, leading to micro-cracking. However, RTW also assists in delaying crack propagation by absorbing strain energy during the load application. As no dedicated SEM fracture surface analysis was conducted, the observed microstructure was sufficient to corroborate the inferred mechanisms. When the reference sample (S0) used no fillers (pure MDI adhesive), its compressive strength was the lowest, measured at 14.128 MPa (Table 3); thus demonstrating a lack of reinforcement and load-carrying capability. Hence, Sample S9 has been identified as the best recycled composite formulation with a balanced contribution of rigidity, toughness, and effective stress transfer. The compressive strength was selected as the primary response parameter for optimisation using RSM. Although apparent compressive modulus values were estimated, detailed stiffness characterization and statistical dispersion analysis were not the main objectives of this study and are considered limitations. These aspects will be addressed in future work through expanded mechanical testing.

**Table 3:** Compressive Strength measurement without and with fillers.

S. No	WRPU (wt%)	RTW (wt%)	WPCB (wt%)	Compressive Strength (MPa)
0	-	-	-	14.128
1	12	6	4	31.284
2	24	6	4	21.480
3	12	12	4	28.140
4	24	12	4	24.324
5	12	6	8	33.392
6	24	6	8	27.029
7	12	12	8	24.914
8	24	12	8	24.727
9	6	9	6	34.310
10	30	9	6	23.355
11	18	3	6	31.102
12	18	15	6	23.154
13	18	9	2	27.149
14	18	9	10	28.688
15	18	9	6	30.659
16	18	9	6	30.659
17	18	9	6	30.659
18	18	9	6	30.659
19	18	9	6	30.659
20	18	9	6	30.659

### Statistical analysis

The statistical analysis was used to determine how the contents of WRPU, RTW and WPCB fillings affect the compression resistance of Flexibond reinforced with filler. To summarize the data compression resistance tests in order to optimize the proportioning of the composite material, design of experiments (DOE), regression modeling and variance analysis (ANOVA) were used. We listed in Table 4 the significant main effects and interactions and we provided a comparative view for how each item affects the compression behavior of the developed foams. The overall regression model exhibited strong statistical significance with an F-value of 17.87 and a P-value of 0.000. This indicates that there is at least one positive impact of the input variables or the interactions on material compression resistance. In the linear analysis using the WRPU, RTW and WPCB contents, we had a combined F-value of 9.29 and a P-value of 0.003. Of these variables it was the WRPU content that had the most (6.86 F-value with a P-value of 0.026). The WPCB content also had a strong effect (8.40 F-value with a P-value of 0.016). The RTW content did not show a significant linear effect, as it had the lowest F-value of 2.77 with a P-value of 0.127. Nonlinear (quadratic) effects - measuring curvature and more complex relationships - were more robust. In total these effects produced an F-value of 8.88 and a P-value of 0.004. The most important single effect across the model was the quadratic term for RTW, which had F-value of 18.36 and a P-value of 0.002. The quadratic effect for WPCB was the second largest, with F-value of 12.04 and a P-value of 0.006. The quadratic term for WRPU was also significant, although the strength of the effect was smaller than the previous two, with an F-value of 6.39 and a P-value of 0.030. Finally, two-way interaction effects were also significant, with an overall total F-value of 8.67 and a P-value of 0.004. The interaction between WRPU and RTW was particularly prominent with an F-value of 12.50 and a P-value of 0.005. An equally prominent interaction was also observed with RTW and WPCB, (F-value of 9.28, P-value of 0.012). The interaction between WRPU and WPCB was less of a factor (F-value 4.22, P-value of 0.067), only just crossing the threshold to be noted as statistically significant. The model error variance was very low, indicated by the adjusted mean square error of 14.797. This means the regression model does a good job of predicting the compressive response. A lack-of-fit F-value was not reported due to there being no pure error in the experimental data, but the model was able to account for more than 90% of the variability in the data, as it could explain 237.959 of 252.756 total sum of squares. Explaining more than 90% of the fit in the experimental data is generally an indication of a good fit to the model. Overall, there were significant effects on the compressive strength of the foam composites by the individual scene input variables and their interactions. The nonlinear (quadratic) effects appeared to have a greater impact than just the linear effects. The quadratic term for RTW had the most influence of all factors evaluated on compressive behavior of the materials.

**Table 4:** ANOVA for Compressive Strength (MPa).

Source	DF	Adj SS	Adj MS	F-Value	*P*-Value
Model	9	237.959	26.4399	17.87	0.000
Linear	3	41.234	13.7447	9.29	0.003
WRPU (wt%)	1	10.144	10.1436	6.86	0.026
RTW (wt%)	1	4.100	4.0996	2.77	0.127
WPCB (wt%)	1	12.436	12.4365	8.40	0.016
Square	3	39.418	13.1392	8.88	0.004
WRPU (wt%) *WRPU (wt%)	1	9.460	9.4595	6.39	0.030
RTW (wt%) *RTW (wt%)	1	27.163	27.1626	18.36	0.002
WPCB (wt%) *WPCB (wt%)	1	17.819	17.8186	12.04	0.006
2-Way Interaction	3	38.472	12.8241	8.67	0.004
WRPU (wt%) *RTW (wt%)	1	18.498	18.4984	12.50	0.005
WRPU (wt%) *WPCB (wt%)	1	6.248	6.2481	4.22	0.067
RTW (wt%) *WPCB (wt%)	1	13.726	13.7257	9.28	0.012
Error	10	14.797	1.4797		
Lack-of-Fit	5	14.797	2.9594	*	*
Pure Error	5	0.000	0.0000		
Total	19	252.756			

**Fig. 6:**
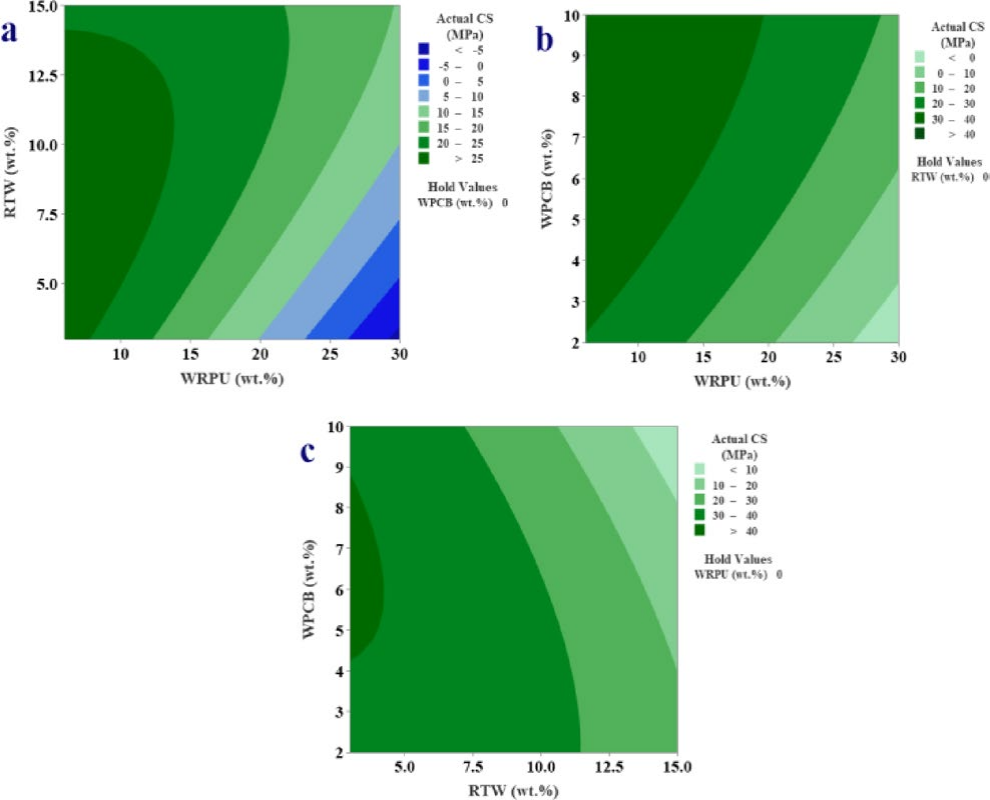
Contour Plots (**a**) WRPU (wt%) vs. RTW (wt%) (**b**) WRPU (wt%) vs. WPCB (wt%) (**c**) RTW (wt%) vs. WPCB (wt%)

Three contour plots were created to examine the impact of weight% (wt%) of WRPU, RTW, and WPCB on compressed power of foam composites. Each contour plot identifies the darkest shades of green to reflect the fields of highest compressed power, while areas with lighter shades of green indicate low strength. The contours indicated in Fig. 6(a) are representative of the influence of WRPU and RTW wt% with WPCB weight fixed; the plots indicated that compressive strength could exceed 25 MPa when WRPU is at or around 6 wt% and RTW is at or around 9 wt%. The plots in Fig. 6(b) represent the effect of WRPU and WPCB with RTW held constant; the plots show that compressive strength may exceed 40 MPa with relatively low WRPU wt% (around 6 wt%) and higher levels of WPCB wt% around 8 wt%, indicating the reinforcing quality of WPCB is evident. The plots in Fig. 6(c) represent the effect of RTW and WPCB with WRPU held constant; the same general observations can be made as 40 MPa compressive strength is again reached for formulations containing approximately 9 wt% RTW and approximately 8 wt% WPCB. Collectively, these contour plots visually represent the two-way interaction effects observed in the ANOVA results (Table 4) and further substantiate the synergistic role that WPCB and RTW are playing in enhancing compressive strength when combined with a lower WRPU matrix content.

**Fig. 7:**
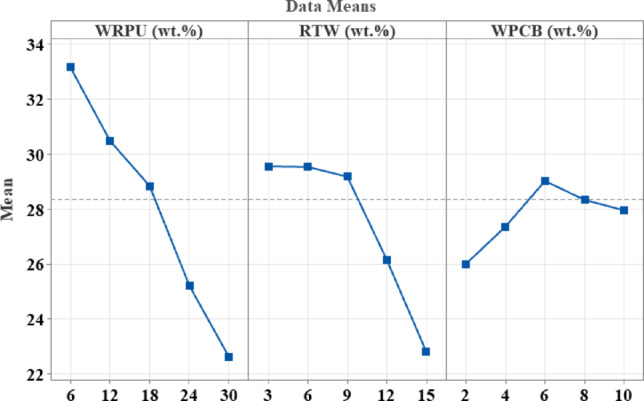
Main Effects Plot for CS (MPa).

The overall impact of combined WRPU, RTW, and WPCB (wt%) on the average compressive strength is shown in Fig. 7. From inspection of this plot, it is clear that WRPU is very influential on compressive strength. It was greatest at 6 wt% WRPU with a value of approximately 34 MPa and it declines in a fairly linear fashion as WRPU continues to increase with respect to compressive strength. After 30 wt% WRPU, the compressive strength is around 22.5 MPa. This aligns with the main linear and quadratic effects identified in the ANOVA. In contrast, RTW is more moderate in impact; compressive strength remains steady around 3 to 9 wt% (approx. 29–29.5 MPa) before steeply declining after 12 wt% to nearly 23 MPa at 15 wt%, supporting its quadratic influence while it was not significant in a linear impact. WPCB in general has a slow rising trend up to 6 wt% where the compressive strength peak around 30 MPa before experiences a small declining trend at 10 wt% similar to WRPU. This meets the statistical significance of WPCB in both linear and quadratic models as shown by ANOVA. Overall, this figure is particularly useful in visualizing the individual influences of each of the three factors and confirming the veracity of effect with model statistical model conclusions.

1 $$\begin{aligned}{\rm{ActualCS}}\left( {{\rm{MPa}}} \right)=&{\rm{29}}{\rm{.21 - 1}}{\rm{.027\,\,\, WRPU}}\left( {{\rm{wt}}{\rm{.\% }}} \right){\rm{ + 1}}{\rm{.306\,\,\, RTW}}\left( {{\rm{wt}}{\rm{.\% }}} \right){\rm{ + 3}}{\rm{.41\,\,\,WPCB}}\left( {{\rm{wt}}{\rm{.\% }}} \right)\\&-{\rm{0}}{\rm{.01704\,\,\,WRPU}}\left( {{\rm{wt}}{\rm{.\% }}} \right){\rm{*WRPU}}\left( {{\rm{wt}}{\rm{.\% }}} \right){\rm{ - 0}}{\rm{.1155\,\,\,RTW}}\left( {{\rm{wt}}{\rm{.\% }}} \right){\rm{*RTW}}\left( {{\rm{wt}}{\rm{.\% }}} \right)\\&-{\rm{ 0}}{\rm{.2105\,\,\,WPCB}}\left( {{\rm{wt}}{\rm{.\% }}} \right){\rm{*WPCB}}\left( {{\rm{wt}}{\rm{.\% }}} \right){\rm{ + 0}}{\rm{.0845\,\,\,WRPU}}\left( {{\rm{wt}}{\rm{.\% }}} \right){\rm{*RTW}}\left( {{\rm{wt}}{\rm{.\% }}} \right)\\&+{\rm{0}}{\rm{.0736\,\,\,WRPU}}\left( {{\rm{wt}}{\rm{.\% }}} \right){\rm{*WPCB}}\left( {{\rm{wt}}{\rm{.\% }}} \right){\rm{ - 0}}{\rm{.2183\,\,\,RTW}}\left( {{\rm{wt}}{\rm{.\% }}} \right){\rm{*WPCB}}\left( {{\rm{wt}}{\rm{.\% }}} \right)\end{aligned}$$

2 $$\rm{Error{\rm{ }}\left( \% \right){\rm{ }} = {\rm{ }}\left( {C{S_{actual}}--{\rm{ }}C{S_{predicted}}} \right){\rm{ }}/{\rm{ }}C{S_{actual}} \times {\rm{ }}100}$$

The regression Eq. (1) defines how the actual compressive strength (MPa) is determined by the three filler components of WRPU RTW and WPCB weight% along with their individual and interaction effects. The intercept of the regression model is 29.21 MPa which tells us what compressive strength would be predicted at zero filler content. The coefficients for the linear term show that the positive linear relationship for WRPU will result in a loss of compressive strength equal to 1.027 MPa per unit increase in WRPU, the relationships for RTW and WPCB result in increases of compressive strength of 1.306 MPa and 3.41 MPa respectively. The coefficients for quadratic components suggest there are non-linear relationships because RTW and WPCB values will see diminishing compressive strength values at high concentrations due to the extreme downward curvature of WPCB. The WRPU*RTW and WRPU*WPCB interaction terms clearly show some minor positive increases in compressive strength but the RTW*WPCB interaction has negative effects decreasing compressive strength with larger quantities. Overall, this regression model explained how each filler affects strength individually and when working together how they affect strength. The compressive strength values from this equation are shown in Table 5 with their respective actual values and error percentages calculated using Eq. (2).

**Table 5:** Actual vs. Predicted CS with Error %.

S.No	WRPU (wt%)	RTW (wt%)	WPCB (wt%)	Actual Compressive Strength (MPa)	Predicted Compressive Strength (MPa)	Residual	Error (%)
1	12	6	4	31.284	32.760	-1.48	-4.718
2	24	6	4	21.480	22.690	-1.21	-5.638
3	12	12	4	28.140	28.970	-0.83	-2.939
4	24	12	4	24.324	24.980	-0.66	-2.707
5	12	6	8	33.392	34.590	-1.20	-3.586
6	24	6	8	27.029	28.050	-1.02	-3.792
7	12	12	8	24.914	25.560	-0.64	-2.582
8	24	12	8	24.727	25.110	-0.38	-1.529
9	6	9	6	34.310	33.140	1.17	3.412
10	30	9	6	23.355	22.620	0.74	3.149
11	18	3	6	31.102	29.550	1.56	5.004
12	18	15	6	23.154	22.800	0.35	1.511
13	18	9	2	27.149	25.990	1.16	4.273
14	18	9	10	28.688	27.940	0.75	2.603
15	18	9	6	30.659	30.330	0.33	1.063
16	18	9	6	30.659	30.330	0.33	1.063
17	18	9	6	30.659	30.330	0.33	1.063
18	18	9	6	30.659	30.330	0.33	1.063
19	18	9	6	30.659	30.330	0.33	1.063
20	18	9	6	30.659	30.330	0.33	1.063

**Fig. 8:**
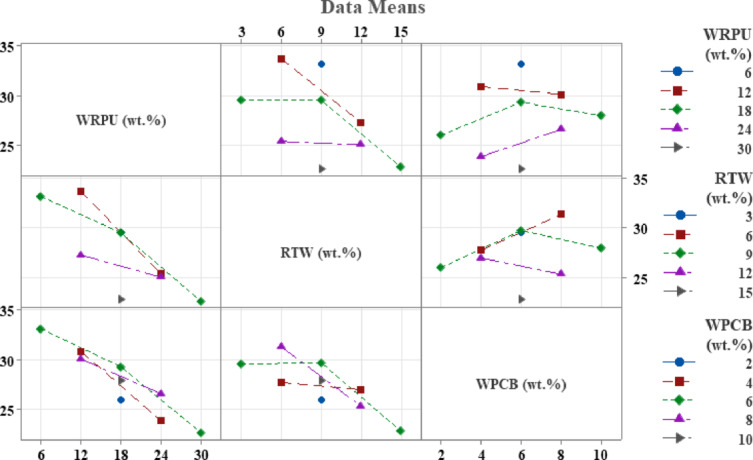
Interaction Plots for Compressive Strength.

The three main factors, WRPU, RTW, and WPCB, and their interaction effects on the compressive strength of the foam samples are illustrated in Fig. 8. Each of the subplots show the interaction between two factors while holding the third factor constant at the various levels available. The trends show that as WRPU increases, the interaction with compressive strength shows considerably different trends based upon the level of RTW and WPCB. In all case, an appreciable reduction in compressive strength is evident with rising levels of WRPU. Consistent with the findings in the regression modelling, there are substantial negative linear and quadratic effects that were distinguished, provided that WRPU is ≤ 30%, compressive strength will be acceptable. RTW had a notably lower interaction with both WRPU and WPCB than WRPU did and could only be described as existing at mild levels of practical significance. While lower than 30% WRPU was observed, the relationship with RTW could be suggested as unchanged, however as a greater amount of WRPU was associated with the more reliable reduction in compressive strength experienced with an increasing trend of RTW. As before, WPCB produced the most overall positive interaction effect, noting that the positive increases in strength were considerable at lower WRPU and moderate levels of RTW. Meaning that assuming these two factors are combined in a favorable manner there is a synergistic contribution to the tensile strength exhibited in the samples through the combination of the two fillers. The trends of the interaction plots, which were non-parallel with some cross-over points, are indicative of two-way interaction effects of note. These interactions once more show what a predictive model is trying to explain. Such identification suggests the potential use of fillers in combination with others more favorably, but also, there was a clear schema for flexibility to adjust or ascertain new combinations in pursuit of refining compressive strength in the foam samples.

**Fig. 9:**
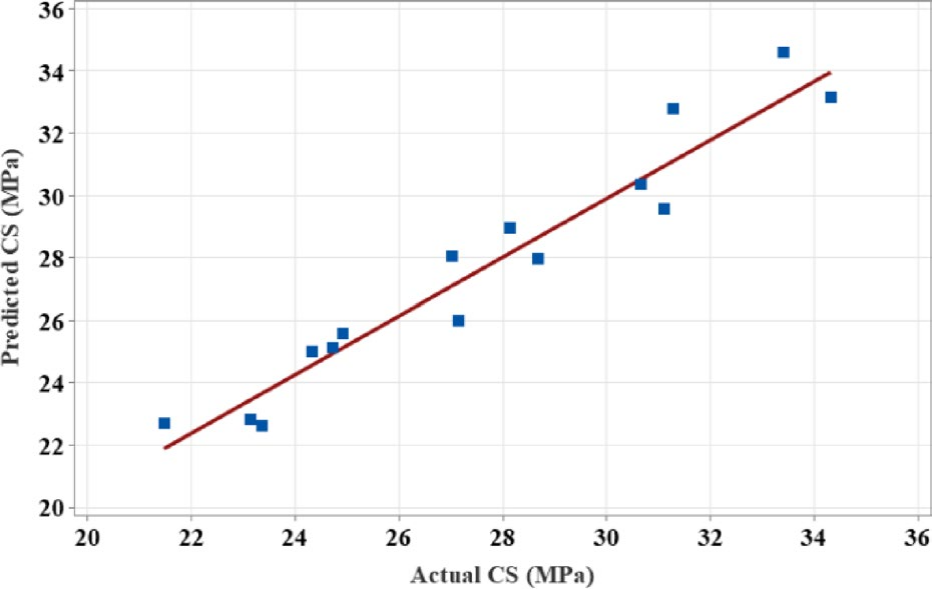
Scatter Plot of Actual CS (MPa) vs. Predicted CS (MPa).

Figure 9 shows the comparison of the actual and predicted compressive strength values based on strong linear regression with high R^2^ values, indicating that Eq. (1) produced from the response surface methodology with statistically reliable results; therefore, it is appropriate to predict compressive strength from many filler combinations. The closeness of the predicted and experimental results illustrates the effectiveness of the predictive model for formulation optimization to achieve better mechanical performance.

The optimal solution shown in Table 6 displays the most effective formulation for creating a composite material that produces the highest compressive strength. The combination that occurred at the optimal solution consisted of 6% WRPU (wt%), 3% RTW (wt%), and 7.57576% WPCB (wt%). The actual compressive strength achieved for this combination was 38.987 MPa. Table 6 also contains the desirability values for the composite, which was also a composite desirability value of 1, indicating that this formulation meets all target conditions and was extremely desirable. This result provides that great practical reference for the optimal choice of fillers to create foam composites with great compressive strengths.

**Table 6:** Optimum wt% of WRPU, RTW, and WPCB.

Solution	WRPU (wt%)	RTW (wt%)	WPCB (wt%)	Compression Strength (MPa) Fit	Composite Desirability
1	6	3	7.57576	38.987	1

**Fig. 10:**
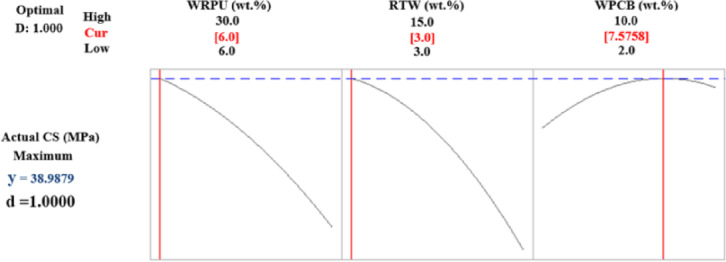
Optimum Curvature of CS (MPa).

Figure 10 shows the response surface optimization of compressive strength, and the weight percentages of WRPU, RTW, and WPCB that were determined to be optimal. This also graphically verifies the settings that would produce the maximum response, and it provides a visual verification for the optimal solution from the model in Table 6.

### Confirmation of sample composition and results

The sample tested for confirmation testing contained 6 wt% WRPU with 3 wt% RTW and 7.57576 wt% WPCB as the optimal formulation. The experimental tests were carried out to confirm the regression model’s predictive values. The measured compressive strength was 38.987 MPa, but the expected value for this formulation was 38.207 MPa, with 2% error. The small difference is a clear indication of the model’s accuracy, and it does indicate that it will produce values for compressive strength. All necessary values are listed in Table 7.

**Table 7:** Confirmation sample with error percentage on experimental results.

Solution	WRPU (wt%)	RTW (wt%)	WPCB (wt%)	Predicted CS (MPa)	Experimental CS (MPa)	Error %
1	6	3	7.57576	38.987	38.207	2%

### Simulation results

**Fig. 11:**
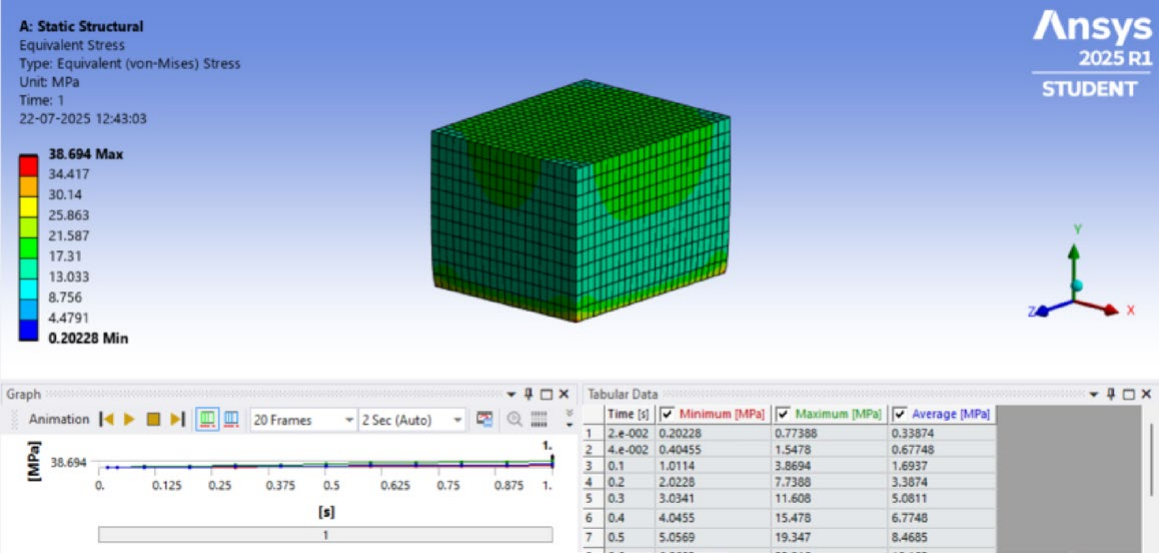
Simulated geometry in ANSYS workbench.

Numerical simulation was conducted in ANSYS Workbench in order to verify the compressive strength of the optimized composite specimens. A cube of 30 × 30 × 30 mm was created in Ansys discovery to replicate the physical sample that was tested. The material was created using experimental figures to define, including Young’s Modulus of 140 MPa and Poisson’s Ratio of 0.26. Based on these figures, ANSYS determined the Shear Modulus automatically as 55.556 MPa and Bulk Modulus as 97.222 MPa. The bottom face of the cube was constrained, and the top face was displaced under a displacement-controlled load in the negative y-axis direction to simulate a typical uniaxial compression test being conducted under the UTM (universal testing machine). Automatic middle was applied, with restraint applied to the same sizes as on the sample in length and breadth to further define the mesh of the upper and lower faces of the sample in stress points of interest. The model was evaluated for total deformation and equivalent (von Mises) stress, with a compressive strength of 38.694 MPa, which has a 0.75% error from the experimental value of compressive strength (38.987 MPa). It will firmly imply that the simulation model is valid and accurate. All values of interest can be found in Table 8. The simulated sample can be seen in Fig. 11. The intent of the numerical simulation is not to replicate the actual composite microstructure, but rather to validate the experimental trend in a simplified manner. The model does not incorporate filler distributions, particle morphologies, or interfacial bonding effects; however, it does provide qualitative knowledge about the distribution of stress and the general mechanical response. Limitations of the model have been established. Further work will be directed towards including multiphase simulations and using the composite microstructure for use in future simulations.

**Table 8:** Confirmation sample with error percentage on simulation results.

Solution	WRPU (wt%)	RTW (wt%)	WPCB (wt%)	Predicted CS (MPa)	Simulation CS results (MPa)	Error %
1	6	3	7.57576	38.987	38.694	0.75%

## Conclusion

This research used waste rigid polyurethane foam (WRPU), recycled tire rubber (RTW), and waste printed circuit board (WPCB) as fillers to create a durable and high-strength composite that is both environmentally friendly and utility. The composite’s ideal formulation was determined through response surface methodology, which revealed that the best combination would be 6 wt% WRPU, 3 wt% RTW and 7.576 wt% WPCB at 38.987 MPa compressive strength. Testing verified that this combination produced a sample with an average compressive strength of 38.207 MPa (an error rate of only 2.00%). Finite Element Analysis results generated using ANSYS also supported the theoretical value of 38.694 MPa (an error rate of 0.75%) proved the accuracy of the statistical modelling approach to the development of composite materials from waste sources. The composite material is well suited for applications where relatively high mechanical loads are applied to parts that lack significant structural support, such as dashboard carriers, under-seat platforms, structures supporting cargo flooring, HVAC brackets, or inner door panel reinforcements. As such, they will serve as excellent examples of automotive parts that require moderate/high strength, weight savings, and dimensional accuracy as primary functional characteristics. The strength and weight properties of polymer composites are consistent with the automotive industry’s trends toward modularization of interior designs and integration of parts for ease of manufacturing, allowing for the combination of multiple parts into a single unit while providing adequate safety levels.

Recycling WRPU, RTW, and WPCB helps the industry meet increasing environmental and regulatory pressures associated with circularity in vehicle use. The use of composites enables OEMs to utilize more recycled content with no impact on mechanical performance. Composite materials are especially well suited for electric or lightweight vehicle designs, where every gram saved or sustainable material used adds directly to the system’s efficiency or range, or fulfills regulatory requirements. As a result, the composite serves as both a technical platform as well as enabling the transition to next-generation sustainable automotive manufacturing. The limitations of variable materials and the need for scalability should also be addressed. One significant limitation to the valorization process of waste streams is the inherent variability in both the physical and chemical characteristics of recycling feedstocks (RTW and WPCB), which can impact on batch-to-batch uniformity. Achieving applicability of this new technology on an industrial scale will require implementing rigorous sorting and pre-processing standards to ensure filler quality consistency. Nevertheless, the composite currently developed has a higher compressive strength-to-weight ratio and cost-efficiency than existing PU systems, despite variability. Future developmental research will focus on eliminating variability and testing for dynamic fatigue properties, to complete validation for mass production.

## Data Availability

Data will be made available on request.
